# Topologically Protected Photovoltaics in Bi Nanoribbons

**DOI:** 10.1021/acs.nanolett.4c01277

**Published:** 2024-05-28

**Authors:** Alejandro José Uría-Álvarez, Juan José Palacios

**Affiliations:** Departamento de Física de la Materia Condensada, Condensed Matter Physics Center (IFIMAC), and Instituto Nicolás Cabrera (INC), Universidad Autónoma de Madrid, Cantoblanco 28049, Madrid, Spain

**Keywords:** Photovoltaics, Topological insulator, Exciton, Optics, Two-dimensional materials

## Abstract

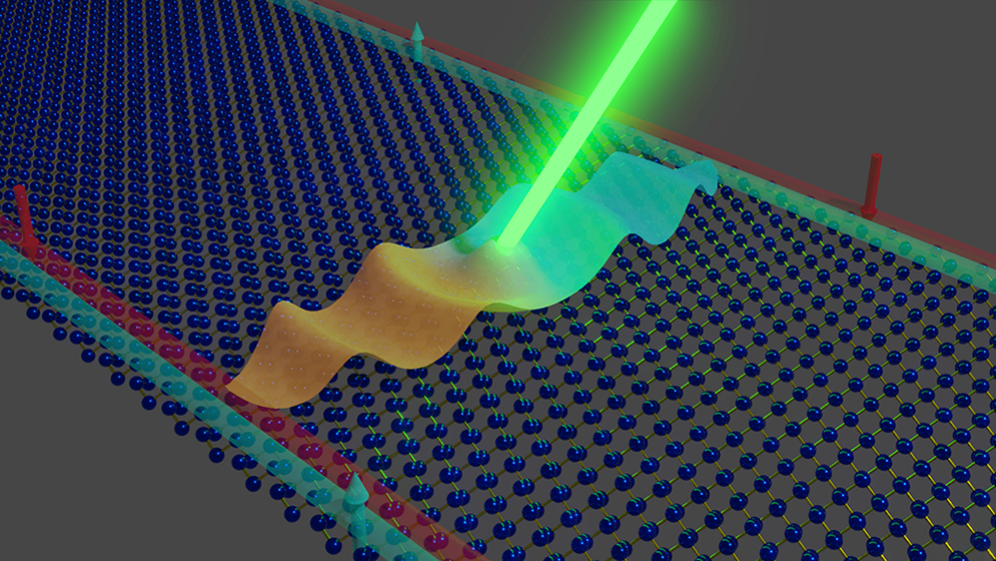

Photovoltaic efficiency in solar cells is hindered by many unwanted effects. Radiative channels (emission of photons) sometimes mediated by nonradiative ones (emission of phonons) are principally responsible for the decrease in exciton population before charge separation can take place. One such mechanism is electron–hole recombination at surfaces or defects where the in-gap edge states serve as the nonradiative channels. In topological insulators (TIs), which are rarely explored from an optoelectronics standpoint, we show that their characteristic surface states constitute a nonradiative decay channel that can be exploited to generate a protected photovoltaic current. Focusing on two-dimensional TIs, and specifically for illustration purposes on a Bi(111) monolayer, we obtain the transition rates from the bulk excitons to the edge states. By breaking the appropriate symmetries of the system, one can induce an edge charge accumulation and edge currents under illumination, demonstrating the potential of TI nanoribbons for photovoltaics.

The creation of pairs of a free electron and a hole may suffice to broadly explain the optical conductivity of insulators and semiconductors, but in general, bound electron–hole (e–h) pairs or excitons also play a non-negligible role.^1−3^ This is particularly true in two-dimensional (2D) crystals, where excitons become tightly bound due to the strong confinement and low screening. These include, for instance, hexagonal boron nitride^4,5^ or transition-metal dichalcogenides, which have been extensively studied in this regard.^6−8^ Additionally, it has also been shown that nonlinear phenomena such as high-harmonic generation^9^ and bulk photovoltaic effects^10,11^ can be greatly enhanced in 2D crystals.^12−15^ This, together with the high tunability of the atomic structure through strain^16^ and the ability to select excitations based on the light polarization, renders 2D optoelectronics as a very active field from both fundamental and technological perspectives.^17^ Of particular interest is light–energy conversion in the form of photocurrent generation, on which solar cell devices are based. The formation of bound e–h pairs and their subsequent separation is the most common source of photocurrent generation. In conventional solar cells, based on p–n junctions, this is achieved with the built-in electric field in the depletion zone, which separates the free charge carriers generating a chemical potential difference or a current depending on the circuit scheme. Since the efficiency of conventional cells is constrained by the Shockley–Queisser limit,^18^ alternative dissociation mechanisms have been proposed as in multijunction cells^19^ or in excitonic solar cells, where a bound electron–hole pair is formed and diffuses to an interface where the charge separation takes place.^20,21^

Topological insulators (TIs), on the other hand, have garnered significant attention in recent years due to their potential for use in spintronic devices, among other more fundamental reasons.^22^ However, there has been relatively little study of TIs from an optical perspective.^23−32^ Only recently, for instance, the exciton spectrum in Bi_2_Se_3_ was shown to exhibit topological properties.^33^

While there are works addressing the role of trivial edge states in the dissociation of excitons in semiconductors,^34−38^ the interaction between bulk excitons in TIs and their topologically protected edge states remains largely unexplored. One recent work studies the interplay between bulk and topological states in the formation of excitons in Bi_2_Se_3_ and how these affect the optical response of the TI.^39^

According to Fermi’s golden rule, an exciton is expected to decay elastically into a continuum of states in the presence of a given coupling. Excitons lie within the energy gap, and in a trivial insulator, there are typically no pure electronic excitations accessible for the exciton to decay into. In general, the usual dissociation channels would be radiative (photons) or nonradiative (phonons) recombination.^40,41^ Topological insulators, instead, always present edge states connecting the valence and conduction bands,^42^ meaning that in addition to light emission, the exciton can decay into e–h pairs formed by edge states. Our principal observation here is that, for sufficiently narrow 2D TI systems (TI ribbons), the electron and hole can also decay onto opposite edges, resulting in a charge separation and eventually in a photovoltaic current.

A purely electronic exciton decay can take place in the form of a noninteracting e–h pair where both constituents are located on the same edge or on opposite edges, which may result in charge transportation since edge electrons and holes typically have finite velocity. Due to time-reversal invariance, however, there is a *k* ↔ −*k* symmetry in the electronic bands (see Figure 2b), meaning that the e–h pair can be equally located at either *k* or −*k*, preventing such a possibility for both inter- and intraedge processes. On top of time-reversal invariance, note also that the system may possess inversion symmetry, forcing any current appearing on one edge to be canceled by the one appearing on the opposite one.

**Figure 1:**
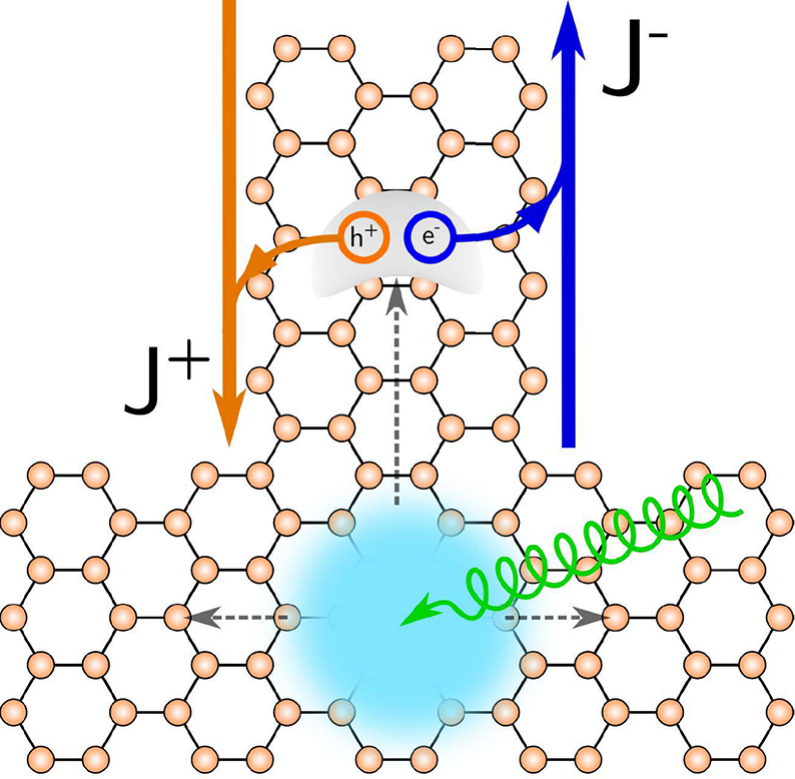
Schematic representation of the proposed mechanism. Device where an exciton wave packet is created at the bulk of the sample, where it will diffuse in any direction. Excitons entering the top ribbon present a finite momentum *Q*, giving rise to an out-of-equilibrium edge carrier population with nonzero momentum and velocity, thus forming a topologically protected current.

**Figure 2:**
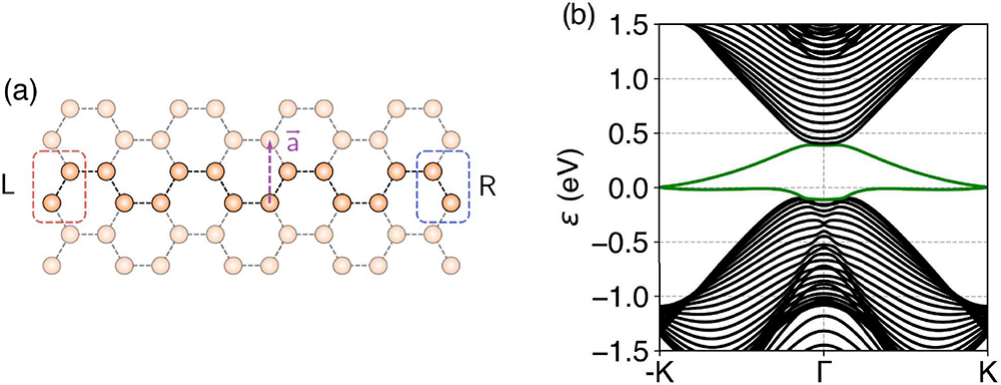
(a) Bi(111) zigzag nanoribbon where the dissociation process takes place. The highlighted atoms denote the unit cell, and a⃗ is the Bravais vector. The edge atoms are identified with the rectangles and labeled as *L* (left) or *R* (right). We introduce onsite energies on the left edge to split the topological edge bands. (b) Band structure of a Bi(111) zigzag ribbon for *N* = 20, with the edge bands highlighted in green.

Even if a priori one is unable to generate current in the presence of time-reversal symmetry, it is still possible to generate a charge imbalance between the edges. Consider that we introduce an asymmetry between the edges via an electric field applied in the direction perpendicular to the infinite edges or simply by some asymmetric termination. The latter is implemented in the following Hamiltonian
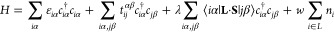
1where the first three terms correspond to *H*_0_, which is a Slater–Koster tight-binding model of a ribbon of Bi(111),^44^ known to be a topological insulator.^45−48^ The last term is the edge offset potential. In particular, we work with a zigzag termination.^46,49^ The width of the ribbon is given by *N*, which is the number of dimers in the ribbon, taken to be even. In Figure 2(a), we show an example unit cell of the Bi(111) ribbon, and we identify the atoms as left (*L*) and right (*R*). On the left ones, we introduce additional onsite energies corresponding to the edge offset to split the edge bands. Then, as long as the perturbation does not close the bulk gap, the edge bands will split, as schematically shown in Figure 3(b). The splitting is expected to produce a different transition rate depending on whether the e–h pair is localized on the left–right boundaries, respectively, or the right–left ones.

**Figure 3:**
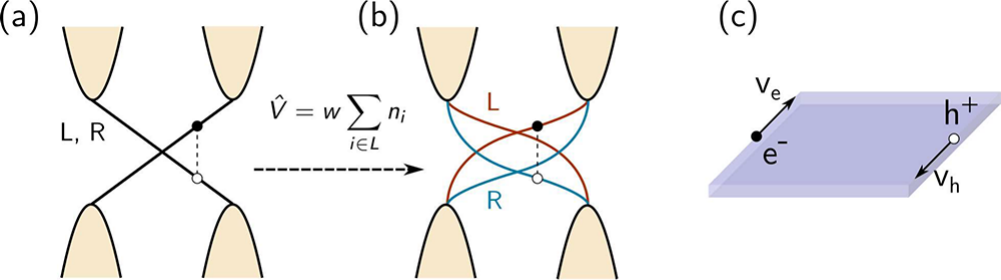
Splitting of the edge bands. (a) For a topological insulator with inversion symmetry, the edge bands of both sides are degenerate, resulting in identical rates for intraedge and interedge transitions. (b) The introduction of an edge offset potential allows the splitting of the edge bands, producing a distinction among the different transitions. (c) For each edge e–h pair, we can determine its total velocity as *v*_e–h_ = *v*_e_ – *v*_h_(43) to establish whether it carries current. For the pair drawn in (b), we observe that *v*_e_ > 0 and *v*_h_ < 0, meaning that *v*_e–h_ = *v*_e_ – *v*_h_ > 0. (See the Supporting Information for the definition of the velocity.)

In addition to charge accumulation, current generation is also possible if time-reversal symmetry is broken. This may occur by the selective population of excitons with nonzero *Q*, avoiding their time-reversal partners with opposite momentum. One possible way to achieve this is shown in Figure 1, where an exciton wave packet is created in the bulk of the sample. This packet is generically described by a momentum distribution |*X*⟩ = *∫ d***Q** *f*(**Q**)|*X*(**Q**)⟩. Since excitons with finite momentum also have finite velocity, some of them will propagate into the top ribbon where the edge offset is present. This populates the ribbon with excitons with finite *Q* but not their time-reversal companions. From the dissociation of these excitons into interedge electron–hole pairs, we expect to generate a topologically protected photocurrent.

To test these hypothesis, we need to evaluate the transition rate from the exciton to each one of the possible electron–hole pairs. Instead of using the band number, we denote each band by its location or edge index, *R* (right) and *L* (left). Thus, for instance, an electron and a hole located on the opposite edges with momentum *k* would be |*L*, *R*, *k*⟩. With this notation, we want to evaluate the following transition rates
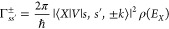
2where *s*, *s*′ ∈ {*R*, *L*} denotes the edge where the electron, hole are localized, respectively, ρ is the density of states of the final continuum of states, namely, the edge e–h pairs, and *V* is the electrostatic interaction. *E*_*X*_ is the energy of the exciton state, defined as the energy of the state relative to the Fermi sea. The initial exciton |*X*⟩ is taken as the *bulk* ground-state exciton
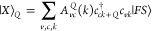
3which is a superposition of electron–hole pairs between any conduction (*c*) and valence (*v*) bands, *excluding the edge bands*. |*FS*⟩ denotes the Fermi sea, and the coefficients  which determine the exciton states are obtained by solving the Bethe–Salpeter equation.^50−53^ Specifically, we assume that all orbitals are point-like, which greatly simplifies the calculation of the exciton spectrum.^54−57^ Regarding screening, we use the Rytova–Keldysh potential.^58−61^ The edge e–h pair is defined as
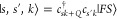
4where  creates a conduction electron such that it is located at side *s* with the specified momentum. The same is done with *c*_*s*′*k*_ for the valence hole.

*k* is chosen such that, given *s*, *s*′, the corresponding e–h pair has the same energy as the exciton. In the case of degeneracies, the rates are obtained by summing over all of the degenerate states. The sign of *k* must also be specified since there are two possibilities and, in principle, transitions can be asymmetric in ±*k*. When the inversion symmetry is removed by the edge offset potential *w*, e.g., at the left boundary, the edge bands, as shown in Figure 4(a), are split. We expect now that the interedge transition rates Γ_*RL*_ and Γ_*LR*_ will be different as the interedge e–h pairs correspond to different |*k*| points (see Figure 4(a)), producing an interedge charge imbalance in the material. This mechanism would compete with intraedge transitions Γ_*RR*_ and Γ_*LL*_, where the electron and hole eventually recombine on the same edge. The intraedge rates serve then as the baseline to estimate the efficiency of the effect.

**Figure 4:**
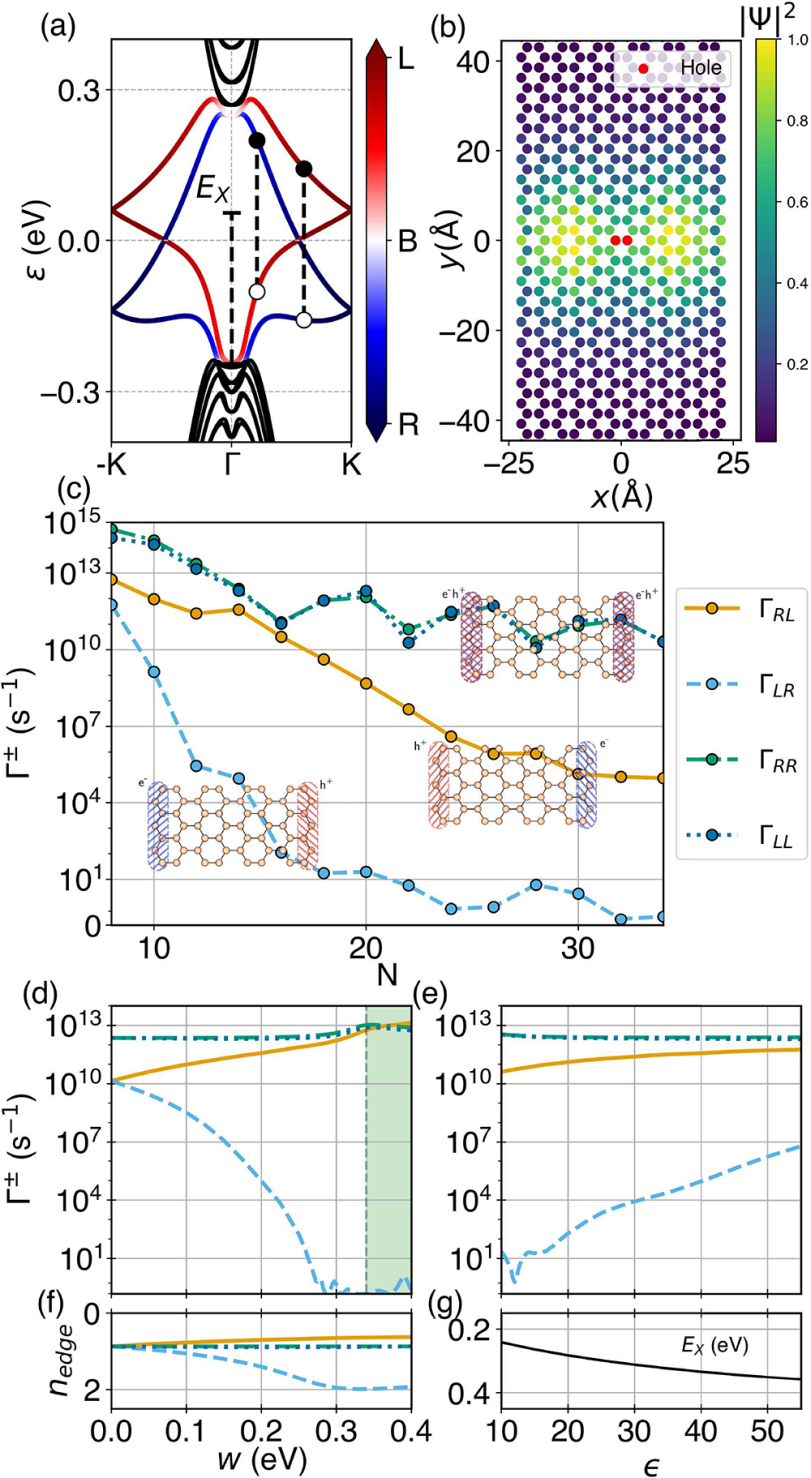
Transitions at *Q* = 0. (a) Band structure of the Bi(111) ribbon for *N* = 20 and *w* = 0.2 eV. The edge bands are colored according to the electronic occupation at the edges of the ribbon. (b) Real-space electronic density probability of the ground-state exciton for *N* = 12. (c) Transition rates of the ground-state exciton to the different edge electron–hole pairs as a function of the width of the ribbon *N* for *w* = 0.2 eV. (d, f) Transition rates and edge occupation as a function of the edge offset potential *w* for *N* = 14. (e, g) Transition rates and ground-state exciton energy as a function of the dielectric constant ε for *N* = 14. (c, d, e, and f) share the same legend.

Regarding the transition rates for *Q* = 0, because of time-reversal symmetry, the transition rates are symmetric in +*k* ↔ −*k* (i.e.,  for *Q* = 0 excitons) (see proof in the Supporting Information). The rates in the presence of an onsite potential (*w* = 0.2 eV) as a function of the width of the ribbon *N* are shown in Figure 4c. In general, as expected, the interedge rates decay faster as a function of *N* than the intraedge ones, with  being several orders of magnitude higher than  for *N* ≈ 10–30.

Notably, for intermediate widths (*N* ≈ 12–16),  turns out to be comparable to intraedge rates. This can be attributed, in part, to the peculiar real-space electronic probability density of the exciton, which exhibits p-like character, as shown Figure 4(b). Moreover, it is possible to tune the rates to enhance the interedge/intraedge ratio. In Figure 4(d), we show the effect of modifying the edge onsite potential *w*. For *w* = 0, there is no charge imbalance since both interedge rates are equal. As we increase the potential, one rate  becomes enhanced as it comes closer to the intraedge rates, while the other  decreases. The effect of the onsite potential approximately splits the edge bands by the same value *w*. Therefore, as we increase *w*, the corresponding edge pairs become increasingly more distant in |*k*|. In Figure 4(a), we see that those involved in  get pushed to the high-symmetry point *K*, where the wave functions are fully localized on the edge. On the other hand, for , the e–h pairs involved get closer to Γ (*k* = 0), where the functions have a stronger bulk component. Therefore, it is possible to improve the interedge/intraedge ratio by tuning the localization of the e–h pairs on the edge, as seen in Figure 4(d). If the edge bands become too far apart, then some of the electron–hole pairs start localizing at different bands, which we indicate with the green region.

A similar discussion can be carried out with the dielectric constants of the system. We focus on the dielectric constant of the material ϵ, although the same arguments apply to the substrate constant ϵ_*s*_. Tuning ϵ produces a change in the exciton energy, which will result in a transition to pairs with different *k*, as shown in Figure 4(e,g). In this case, the specific behavior will be dependent on the form of the bands. Similar to the onsite potential, changing the exciton energy drastically could result in pairs hosted in a different set of bands from before, although this is not the case for the range of values considered.

With respect to transition rates for *Q* ≠ 0, next we consider the transition rates for excitons with finite momentum *Q*. As for *Q* = 0 excitons, the edge charge accumulation will still be present as long as we keep the edge offset term finite (we again set a fixed value of *w* = 0.2 eV). Now, the main difference with respect to the rates for *Q* = 0 excitons comes from the asymmetry in *k*. Since the initial exciton is not time-reversal-invariant (as it has finite momentum *Q*), all of the transition rates  will be different. (Figure 5(a) shows the interedge processes schematically.) Both intraedge and interedge pairs can carry a net current since they have a finite total velocity *v*_e–h_(*k*) ≠ 0, but now there will be no exact cancellation between *k* and −*k* pairs.

**Figure 5:**
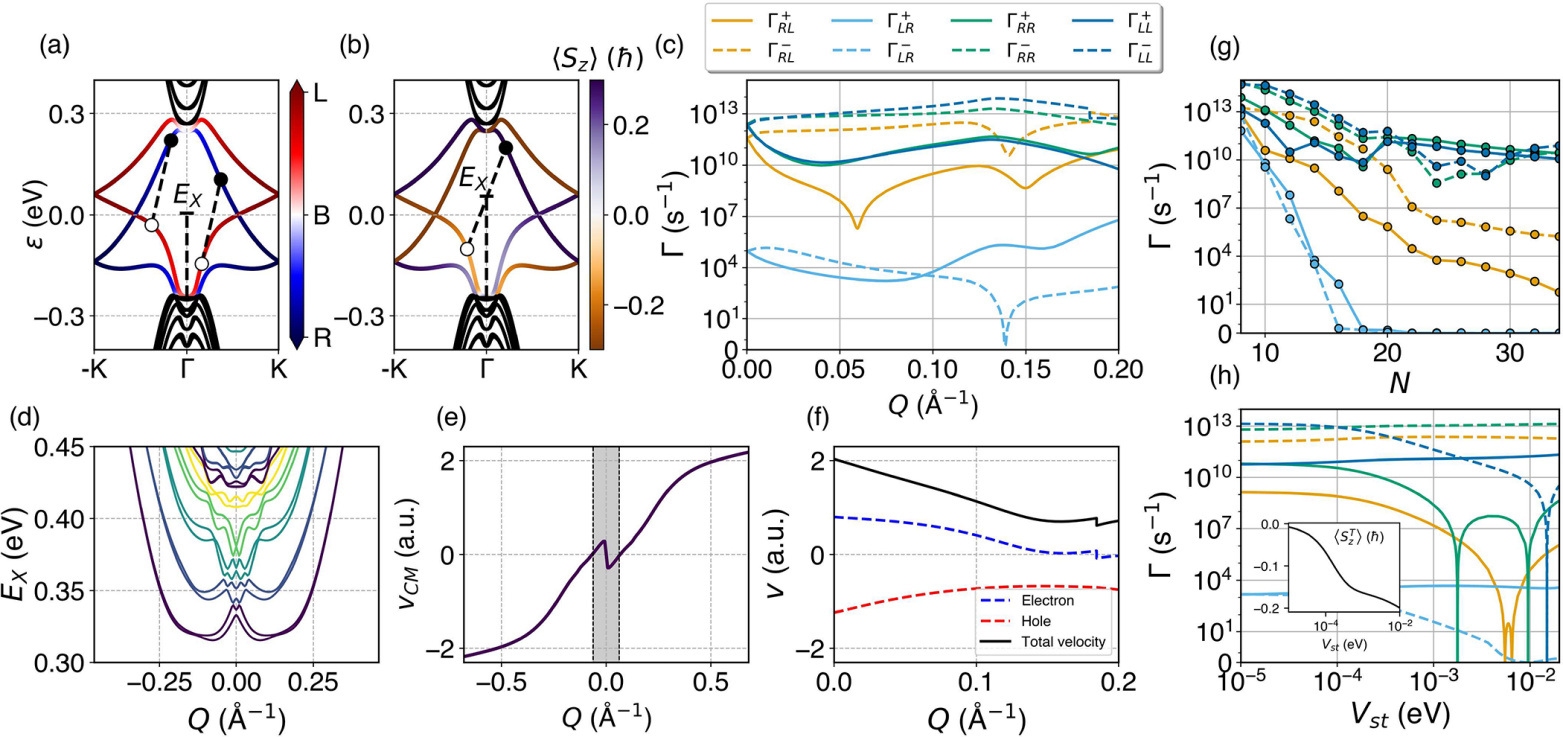
Transitions at finite *Q*. (a, b) Band structure of the Bi(111) ribbon for *N* = 20 and *w* = 0.2 eV. The first one shows the edge occupation of the bands, and the second one shows the average spin projection  of the bands. (c) Transition rates of the ground-state exciton as a function of *Q* for *N* = 14. (d) Low-energy exciton band structure. Each color corresponds to four excitonic states in total. (e) Center-of-mass velocity of the ground-state exciton. The shadowed region denotes the fraction of excitons that do not contribute to the formation of an edge current. (f) Velocity *v* = *v*_e_ – *v*_h_ of the relevant electron–hole pair  and of each component individually, for *N* = 14 and *w* = 0.2 eV. (g) Transition rates as a function of *N* for *Q* = 0.1 Å^–1^ and *w* = 0.2 eV. (h) Transition rates as a function of the staggered potential *V*_*st*_ for *N* = 14 and *w* = 0.2 eV. The inset shows the total spin projection of the ground-state exciton, , as a function of the staggered potential.

The results, displayed in Figure 5(c), show the expected behavior: as *Q* becomes nonzero, the ±*k* symmetry of the rates is lifted, namely, . We observe that, for the values of *Q* considered,  and  rates differ by several orders of magnitude, meaning that the charge separation still takes place. We focus our attention again on these interedge rates since electron–hole pairs localized on the same edge are assumed not to contribute to the current as they are prone to recombination (in this case via phonon emission first). One interedge rate  is close in magnitude to the intraedge ones for all of the values of *Q* considered. We also see that  differs by several orders of magnitude from , supporting our hypothesis that an overall edge current can develop in the material since we are inducing an electronic population imbalanced in *k*. For reference, we show in Figure 5(f) the total velocity of the electron–hole pair corresponding to , which is nonzero and positive for the values of *Q* considered. Note that the plot shows values of *Q* only up to 0.2. For higher values of *Q*, the energy of the exciton increases quadratically (see Figure 5(d)), and as a consequence, there are no longer available edge e–h pairs. Also, as *Q* increases, it might happen that either the electron or the hole changes the band where it is hosted, as illustrated in Figure 5(a,b). This produces the discontinuity in the rates and the velocities appearing at *Q* ≈ 0.18.

The fraction of excitons entering the ribbon (see Figure 1) is determined by the velocity of these. We have thus computed the total velocity or center-of-mass velocity of the exciton *v*_X_ as a function of *Q*, as shown in Figure 5(e). Those with *v*_X_ > 0 will enter the ribbon. For a small fraction with *Q* > 0, highlighted by the gray region, the excitons have negative velocity (i.e., they move away from the ribbon). For the fraction of excitons of the highlighted region with negative *Q*, they enter the channel but contribute with currents opposite (due to time reversal) to the ones with *Q*, *v*_X_ > 0. However, from the exciton bands in Figure 5(d), we conclude that it is more likely to have a population of excitons satisfying the latter condition, as it corresponds to a lower energy overall.

As we did for *Q* = 0, in Figure 5(g) we show the behavior of the transition rates as we increase the width of the ribbon for *Q* = 0.1. As expected, the interedge rates decay faster than the intraedge rates. Importantly, up to *N* = 20, the relevant interedge rate  is comparable to the intraedge ones. The opposing rates  become completely suppressed from *N* = 18, enhancing the charge separation. As for the ratio between  and , it appears to be relatively constant for the widths under consideration.

Finally, we show that it is also possible to engineer the rates by further tuning the spin of the exciton. For *Q* = 0, we obtain that  due to the bulk bands being degenerate. However, if the exciton had a finite value of the spin, then we would expect different values for the rates  given that the edge bands also present opposite spin when *k* ↔ −*k*, as shown in Figure 5(b). We can induce this finite spin introducing a sublattice staggered potential that breaks inversion symmetry in the bulk of the material, thereby fully splitting the bulk bands (see the Supporting Information). We show in Figure 5(h) how for *Q* = 0.1 this potential induces a spin in the ground-state exciton (inset) and results in the relevant rates ,  deviating even further from each other. Remarkably, some of the intraedge rates, which may hinder the performance of the device, are strongly suppressed in a wide range of the staggered potential, even becoming zero at particular values.

Since current generation is possible only with *Q* ≠ 0 excitons, we note that radiative recombination will not be present due to the finite exciton momentum. Furthermore, for the *Q* = 0 excitons considered, we observe a vanishingly small oscillator strength corresponding to dark excitons. In fact, the main limiting factor will be the exciton–phonon scattering. For reference, other bidimensional materials show exciton lifetimes due to phonon scattering between 1 and 1000 fs.^62,63^ Assuming similar lifetimes for Bi(111), the relevant rate  is approximately of the same magnitude for all values of *Q* considered. We note that the effect is heavily dependent on the ribbon width, meaning that for wider systems the exciton–phonon scattering will eventually dominate.

We have noted that the edge states of a 2D TI constitute an alternative dissociation path to exciton recombination. To this end, we have fully characterized the exciton spectrum in Bi(111) nanoribbons and have shown that if one introduces an onsite edge potential to split the edge states then one can possibly obtain an edge charge imbalance from the dissociation of excitons into noninteracting edge electron–hole pairs. Additionally, we have shown that if we are able to generate a population of excitons in the ribbon that is not time-reversal-invariant, then an edge current (topologically protected) may develop. Moreover, the corresponding transition rates can be tuned to increase or decrease the strength of the effect. Our estimates (see the Supporting Information) indicate that currents in the μA range can be obtained. The present arguments are not dependent on the specific shape of the bands, and we expect that they can be applied and tested both theoretically and experimentally with other 2D TIs such as Bi_4_Br_4_, which is a room-temperature TI.^64^ The foundation of the effect is not exclusive of the dimensionality and can be trivially extended to three-dimensional TIs. Remarkably, in a recent work the relaxation of photoexcited bulk electrons onto topological surface states in Bi_2_Se_3_ has been reported,^65^ indirectly supporting our claim.

## Supporting Information Available

Expression of the Bethe–Salpeter equation; exciton spectrum in ribbon and bulk Bi(111); expression, properties, and convergence of the transition rates, expressions for the exciton velocity and spin,; transition rates on an armchair ribbon; and an estimation of the generated photocurrent (PDF)
