# Supplementary material for: Topologically Protected Photovoltaics in Bi Nanoribbons
Source: Nano Lett. 2024 May 28;24(22):6651–7. doi: 10.1021/acs.nanolett.4c01277 (PMC11157647; doi:10.1021/acs.nanolett.4c01277)
Supplement: Supplementary file 1 — nl4c01277_si_001.pdf [file nl4c01277_si_001.pdf]

Supporting Information for Topologically protected photovoltaics in Bi nanoribbons

Alejandro José Uría-Álvarez* and Juan José Palacios

Departamento de Física de la Materia Condensada,

Condensed Matter Physics Center (IFIMAC),

and Instituto Nicolás Cabrera (INC),

Universidad Autónoma de Madrid, Cantoblanco 28049, Spain

(Dated: May 17, 2024)

Abstract

We provide additional details regarding the exciton spectrum, the calculation of the transition rates and the expressions used to compute the rest of the quantities in the main text.

* alejandro.uria@uam.es

I. METHODS

The material of choice here is a monolayer of Bi(111), also known as *beta*-bismuthene, which was predicted [1, 2] and observed to be a 2D TI [3, 4]. We use a Slater-Koster tight-binding model which properly describes the electronic structure of Bi(111) [5]. Monolayer Bi(111) appears as a puckered honeycomb lattice and presents two possible standard edge terminations: zigzag and armchair [2]. We consider ribbons with zigzag terminations, defining N as the number of Bi dimers across the unit cell. In particular, we choose N to be even so that the space group of the ribbon is symmorphic [6] to restrict the symmetry discussion to the point group. Nevertheless we do not expect this to be relevant for the calculations forward.

With the material model established, we now turn our attention to the description of excitons. As they are bound electron-hole pairs, one must consider an electrostatic interaction between the electrons of the solid, i.e.:

$$H = \sum_{nk} \varepsilon_{nk} c^\dagger_{nk} c_{nk} + \frac{1}{2} \sum_{ijlm} V_{ijlm} c^\dagger_i c^\dagger_j c_m c_l \tag{1}$$

ε_{nk} denotes the bands of the ribbon, and V_{ijlm} are the matrix elements of the electrostatic interaction. The indices i, j, l, m are short-hand notation for pairs of quantum numbers (n, k), with n the band index and k the crystal momentum, which is an scalar since the system is one-dimensional. The exciton states are then given by a superposition of electron-hole pairs between valence and conduction bands:

$$|X\rangle_Q = \sum_{v,c,k} A^Q_{vc}(k) c^\dagger_{ck+Q} c_{vk} |\Omega\rangle \tag{2}$$

where $|\Omega\rangle$ denotes the Fermi sea, which we assume is the ground state of the system, i.e. we work in the Tamm-Dancoff approximation [7]. Q denotes the center-of-mass or total momentum of the exciton. For the bulk excitons we discuss throughout the article, we restrict v and c to bulk bands, i.e. all bands except for the edge ones (those closing the gap). Then, the problem of determining the exciton coefficients $A^Q_{vc}(k)$ amounts to diagonalizing the total Hamiltonian (1) projected over the sector of single electron-hole pairs, i.e. PHP. Therefore, we need to obtain the matrix elements of the interacting Hamiltonian represented in the basis of electron-hole pairs. Denoting the electron-hole pairs as $|v, c, k, Q\rangle = c^\dagger_{ck+Q} c_{vk} |\Omega\rangle$, the

matrix elements $\langle v, c, k, Q|H|v', c', k', Q\rangle \equiv H^{cc'}_{vv'}(k, k', Q)$ are given by:

$$H^{cc'}_{vv'}(k, k', Q) = (\varepsilon_{ck+Q} - \varepsilon_{ck})\delta_{cc'}\delta_{vv'}\delta_{kk'} - (D - X)^{cc'}_{vv'}(k, k', Q) \tag{3}$$

The D, X terms represent the direct and exchange interaction between the electron and the hole, and are given by some specific matrix elements of the two-body interaction V:

$$\begin{aligned} D^{cc'}_{vv'}(k, k', Q) &= V_{ck+Q,v'k',c'k'+Q,vk} \\ X^{cc'}_{vv'}(k, k', Q) &= V_{ck+Q,v'k',vk,c'k'+Q} \end{aligned} \tag{4}$$

This approach to excitons has been used previously in several works [8–11], since it is a simpler and faster alternative to many-body perturbation theory. To compute the excitons, we need to evaluate explicitly the direct and exchange terms (4) in terms of the tight-binding Bloch states. If $\{C^{nk}_{i\alpha}\}_{i\alpha}$ are the coefficients corresponding to the eigenstate $|nk\rangle$ in the lattice gauge [12], then the interaction matrix elements D can be written as:

$$D_{vc,v'c'}(k, k', Q) = \sum_{ij}\sum_{\alpha\beta}(C^{ck+Q}_{i\alpha})^*(C^{v'k'}_{j\beta})^* C^{c'k'+Q}_{i\alpha} C^{vk}_{j\beta} V_{ij}(k' - k) \tag{5}$$

where

$$V_{ij}(k' - k) = \frac{1}{N}\sum_{\mathbf{R}} e^{i(k'-k)R} V(\mathbf{R} - (\mathbf{t}_j - \mathbf{t}_i)) \tag{6}$$

$\mathbf{R}$ are Bravais vectors, $R = |\mathbf{R}|$ and $\mathbf{t}_i$ are the atomic positions of the motif. V_{ij} denotes a lattice Fourier transform of the potential centered at $\mathbf{t}_j - \mathbf{t}_i$. This expression is obtained evaluating the four-body integral of the interaction in real-space, using the tight-binding approximation (i.e. point-like orbitals) [13]. It is also possible to derive an alternative expression for the interaction matrix elements with the interaction in reciprocal space through its Fourier series [14, 15]. Given that the platform for our excitons is a semi-infinite ribbon, the real-space approach is better suited since it takes into account the finite boundaries. As for the exchange term X, we set $X = 0$ assuming that its contribution is negligible.

For the electrostatic interaction, we use the Rytova-Keldysh potential [16, 17]. This model has gained interest to describe excitons in two-dimensional materials since it includes screening, as opposed to the bare Coulomb interaction. This is particularly relevant since the exact diagonalization approach does not screen the bare interaction, while many-body perturbation theory does, usually through the random-phase approximation [18]. The Rytova-Keldysh potential is given by:

$$V(\mathbf{r}) = \frac{e^2}{8\epsilon_0\bar{\epsilon}r_0}\left[H_0\left(\frac{|\mathbf{r}|}{r_0}\right) - Y_0\left(\frac{|\mathbf{r}|}{r_0}\right)\right] \tag{7}$$

where $\bar{\epsilon}$ is an environmental dielectric constant, given by $\bar{\epsilon} = (\epsilon_s + \epsilon_v)/2$, with ϵ_s, ϵ_v the dielectric constants of the substrate and vacuum respectively. r_0 is a screening length, such that $r_0 = d\epsilon/(\epsilon_s + \epsilon_v)$, with d the height of the layer and ϵ the dielectric constant of the material of interest [19]. H_0, Y_0 are Struve and Bessel functions of second kind respectively. We follow the prescription of [8] to renormalize the divergence of the potential at $r = 0$, by setting $V(0) = V(a)$, a being the lattice parameter of Bi(111). For our monolayer Bi(111), we set the dielectric constant to $\epsilon = 40$, and the environmental one to $\bar{\epsilon} = 2.45$, corresponding to a SiO_2 substrate.

The edge electron-hole pairs are defined as conventional pairs, but instead we specify the boundary rather than the band number:

$$|s, s', k\rangle = c^{\dagger}_{sk+Q} c_{s'k} |GS\rangle \tag{8}$$

where $c^{\dagger}_{sk+Q}$ creates a conduction electron such that it is located at side s with the specified momentum. The analogue is done with $c_{s'k}$ for the valence hole, with the overall restriction that the energy of the pair is the same as that of the exciton. Note that for complex edge bands, there could be several electron-hole pairs verifying the same conditions, and so all of them should be taken into account in the transition rates. The calculation of the transition rates is done with Fermi's Golden rule, which involves the matrix elements of the electrostatic interaction between the exciton and the final edge electron-hole pair. Expanding the exciton into electron-hole pairs, the transition rates can be cast in terms of the direct and exchange terms of eq. (4). Since we neglect the exchange term, the final expression for the rates is:

$$\Gamma^{\pm}_{ss'} = \frac{2\pi}{\hbar} \left| \sum_{v,c,k'} A^{Q}_{vc}(k') D^{sc}_{s'v}(k_e, k', Q) \right|^2 \rho(E_X) \tag{9}$$

The calculation of the velocity is based on the second quantized version of the velocity operator, namely $v = \sum_{ij} v_{ij} c^{\dagger}_i c_j$ where the indices i, j correspond to pairs (n, k). Taking the expected value with the edge electron-hole pair yields the following expression:

$$v_{e-h} \equiv \langle v \rangle_{e-h} = v_{ck+Q,ck+Q} - v_{vk,vk} \tag{10}$$

this is, the total electronic velocity is given by the velocity of the electron minus the velocity of the hole [20]. For the computation of the single-particle velocity elements we refer to [12].

Anagolously, we can compute the total electronic velocity of the exciton:

$$\begin{aligned}
&\langle v\rangle_X = \\
&\sum_{v,c,k} A^Q_{vc}(k)\left[\sum_{c'}(A^Q_{vc'}(k))^* v_{c'c} - \sum_{v'}(A^Q_{v'c}(k))^* v_{vv'}\right] \\
&\equiv \langle v_e\rangle_X - \langle v_h\rangle_X
\end{aligned} \tag{11}$$

Note that as with the electron-hole pair, the velocity of the exciton can be separated into contributions from the electron and the hole separately. In this context, the velocity operator can be rewritten as:

$$v = \sum_{c,c',k} v_{ck,c'k} c^\dagger_{ck} c_{c'k} - \sum_{v,v',k} v_{vk,v'k} c^\dagger_{vk} c_{v'k} \equiv v_e - v_h \tag{12}$$

where we have defined two new operators. This leads to defining the center-of-mass velocity of the exciton as:

$$v_X \equiv v_{CM} = \langle v_e\rangle_X + \langle v_h\rangle_X \tag{13}$$

which is the relevant quantity for determining the propagation direction of the excitons.

All the quantities shown in the article, from the exciton spectrum to the probability densities and the transition rates have been calculated using the Xatu code [13]. For the calculation of excitons we have used $N_v = N_c = 4$, where N_v (N_c) is the number of bulk valence (conduction) bands taken into account, and a minimum of $N_k = 800$ points in the Brillouin zone to ensure convergence of all transition rates.

All the excitons have been computed using $N_v = N_c = 4$ and a minimum of $N_k = 800$, while in some cases the number of k points was even higher to ensure that the lowest magnitude transition rates were properly converged, up to $N_k = 1200$. Here we show additional results relative to the convergence of both the excitons and the transition rates with the number of k points, as well as the effect of the number of bands on the results of the main text.

The exciton energies converges relatively quickly with N_k, requiring around $N_k \sim 200$ to be converged. On the other hand, the number of bands included in the BSE is particularly relevant since we are working with a ribbon, i.e. a semi-infinite system where the previous degree of freedom $k_\perp$ of the periodic system now corresponds to additional bands in the

system, which are more closely packed. Therefore, in principle one should include a number of bands in the ribbon equivalent to the number of k points one would use for the 2D periodic system. However, as we will see, the convergence of transition rates with N_k imposes a computational restriction on the number of bands we can consider, given that the BSE matrix size scales as $N_c N_v N_k$.

When computing the exciton spectrum with $Q = 0$ and without spin-orbit coupling (SOC), we observe a four-fold degeneracy of all energy levels. This is to be expected, since the point group of the ribbon is C_{2h}, whose irreducible representations (irreps) are all of dimension 1. For a spinless system, all states would be non-degenerate, while for a spinful system with neither SOC nor exchange, four-fold degenerate. As we turn on the SOC, the exciton spectrum acquires a fine structure. The character table of the C_{2h} double group shows one-dimensional irreps [21]. However, we observe two-fold degeneracy for some states, in particular for the ground state. This is produced by the presence of inversion symmetry which results in degenerate bands, together with time-reversal symmetry even in presence of spin-orbit coupling. The introduction of the sublattice potential, which breaks inversion symmetry, lifts the quasiparticle band degeneracy and also results in a splitting of the degenerate exciton bands.

The real-space electronic density probability of the excitons, is computed from the following expression:

$$\Psi_X(\mathbf{r}_e, \mathbf{r}_h) = \sum_{v,c,k} A^Q_{vc}(k)\psi_{ck+Q}(\mathbf{r}_e)\psi^*_{vk}(\mathbf{r}_h) \tag{14}$$

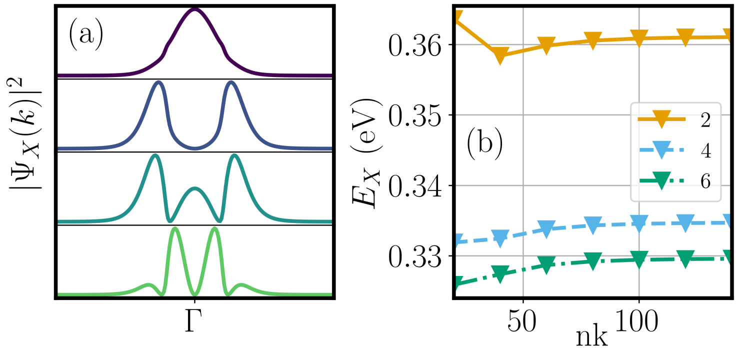

FIG. S1. (a) k probability density of the first four exciton levels for $N_v = N_c = 2$. (b) Convergence of the ground state exciton energy with N_k and the number of bands included for both valence N_v and conduction N_c, i.e. $N_c = N_v = 2, 4$ or 6

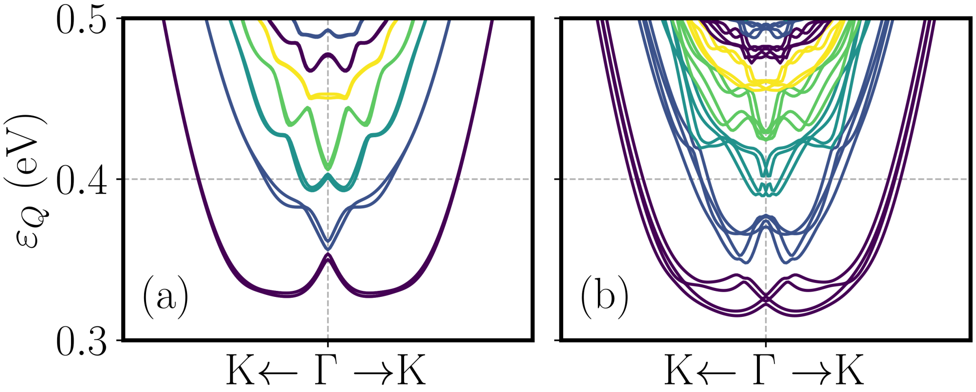

FIG. S2. Exciton band structure as a function of center-of-mass momentum Q, for $N_v = N_c = 2$. (a) Band structure without inversion breaking potential V_{st} and (b) with $V_{st} = 0.1$ eV.

which is evaluated using the tight-binding approximation. In Fig. (S3) we show the wavefunctions with and without SOC. Without SOC we observe the standard hydrogenic spectrum, starting from s states, then p and so on. Instead, for the topologically insulating state the ground state is now p-like along the finite direction, instead of the periodic one. This appears to be an effect of the confinement of the wavefunctions, as the same computation for bulk Bi(111) shows that the ground state for both the trivial and topological phases are s-like.

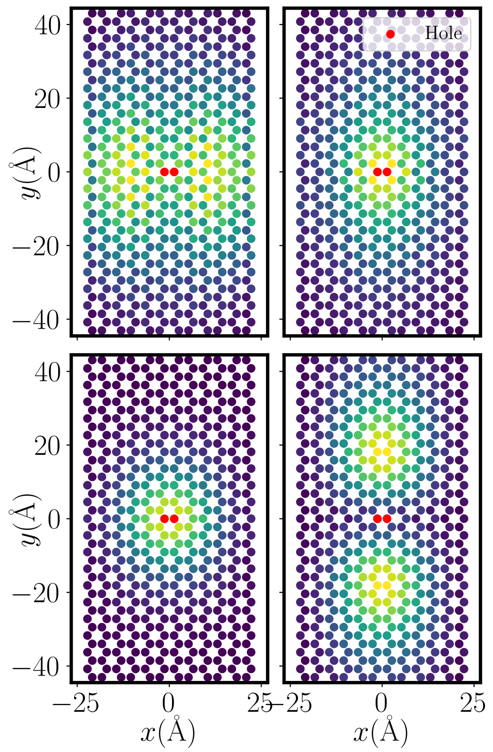

FIG. S3. Real-space electronic probability density of the ground state exciton (left) and first excited state (right). The top row densities correspond to the system with SOC, while the bottom ones are computed without SOC.

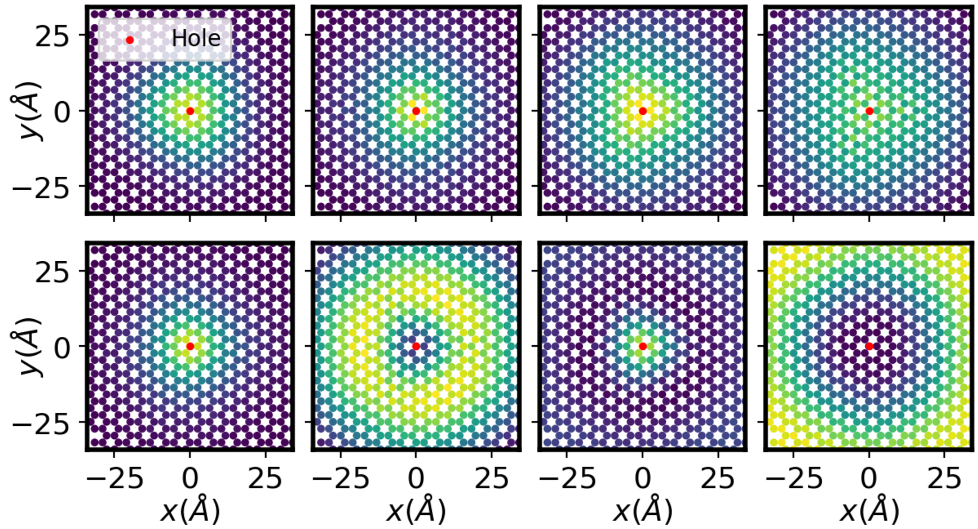

FIG. S4. Bulk real-space electronic density probability of the first four exciton levels, from left to right. Top row corresponds to excitons in Bi(111) with SOC, while the bottom row corresponds to excitons computed without SOC.

As it is shown in the section for $Q \neq 0$ in the main text, we also characterize the total spin projection of the ground state exciton as well as the center of mass velocity of the exciton, which are both calculated the same way. The total spin projection is simply given by the the second quantized version of the S_z operator:

$$S_z = \sum_{i,j} \sigma_{ij} c_i^\dagger c_j \tag{15}$$

where i, j denote pairs of quantum numbers $i \equiv (n, k)$, and $\sigma_{ij} = \langle i|S_z|j\rangle$. Taking the expectation value with an exciton state, one obtains:

$$\langle S_z \rangle = \sum_{v,c,k} A_{vc}^Q(k) \left[\sum_{c'} (A_{vc'}^Q(k))^* \sigma_{c'c} - \sum_{c'} (A_{v'c}^Q(k))^* \sigma_{vv'} \right] \equiv \langle S_z^e \rangle + \langle S_z^h \rangle \tag{16}$$

which can be regarded as the expectation value of the spin taken over the conduction electron plus the expectation value taken over the valence hole. We have defined here new spin operators associated with the electron and hole, respectively:

$$S_z = \sum_{c,c',k} \sigma_{ck,c'k} c_{ck}^\dagger c_{c'k} - \sum_{v,v',k} \sigma_{vk} c_{v'k} c_{v'k}^\dagger \equiv S_z^e + S_z^h, \tag{17}$$

into which S_z can be split.

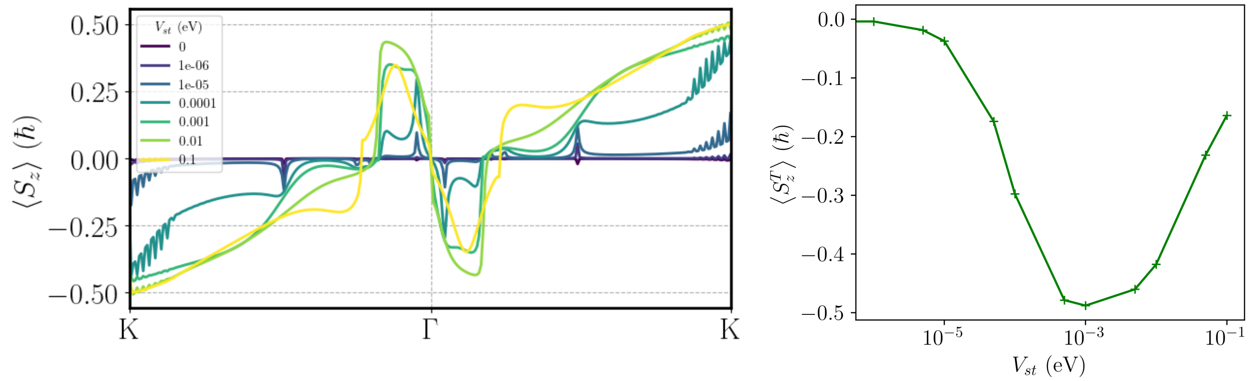

FIG. S5. (Left) Total expected spin projection $\langle S_z^T \rangle$ of the exciton as a function of Q for $N_v = N_c = 2$, $N_k = 200$, for different values of the staggered potential V_{st}. (Right) Total exciton spin $\langle S_z^T \rangle$ for $Q = 0.1$ as a function of the staggered potential V_{st}, for $N_v = N_c = 2$, $N_k = 200$.

Analogously, to compute the total electronic velocity of the exciton, one generically defines the velocity in second quantization:

$$v = \sum_{ij} v_{ij} c_i^\dagger c_j \tag{18}$$

Again, taking the expected value with an exciton states yields an expression analogue to the previous one, now in terms of the velocity matrix elements:

$$\langle v \rangle = \sum_{v,c,k} A^{Q}_{vc}(k) \left[\sum_{c'} (A^{Q}_{vc'}(k))^{*} v_{c'c} - \sum_{c'} (A^{Q}_{v'c}(k))^{*} v_{vv'} \right] \equiv \langle v_e \rangle - \langle v_h \rangle \tag{19}$$

Note that in this case, the minus sign is left out of the definition of the hole velocity, since the velocity of a hole is the same as the velocity of the equivalent electron [20], whereas the spin is the opposite. This means that the total electronic velocity of the exciton, is actually the relative velocity between its components. This leads to defining the center-of-mass velocity of the exciton in the following way:

$$\langle v_{CM} \rangle = \langle v_e \rangle + \langle v_h \rangle \tag{20}$$

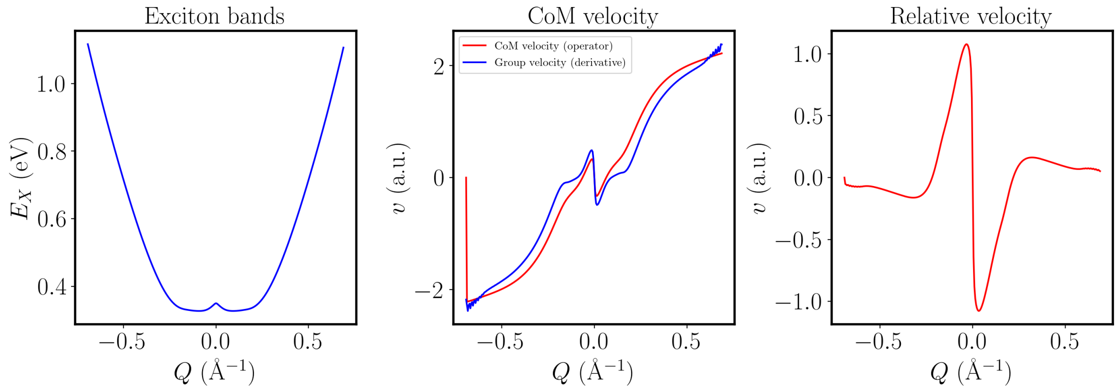

FIG. S6. (Center) Center-of-Mass velocity and (right) relative velocity of the ground state exciton for excitons with $N_v = N_c = 2$, $N_k = 200$. Only a small fraction of the ground state excitons with finite **Q** propagates backwards, due to the characteristic band inversion of topological insulators which produces excitonic bands with the same feature.

Eq. (18) is also used to compute the total velocity of the final edge electron-hole pairs, i.e. to determine whether the final Slater determinants form a current in the material or not. In this case, the total velocity is given simply by $\langle v \rangle = v_{cc} - v_{vv}$, this is, in terms of the diagonal velocity matrix elements. As for how to compute the velocity matrix elements, we refer to [12].

Next, we show how the transition rates converge with the number of k points in the Brilloin zone. From the convergence plots we see that the lower the magnitude of the

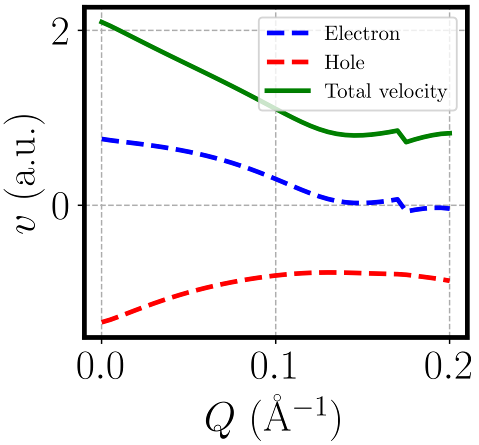

FIG. S7. Total velocity of the final electron-hole pair, and velocity of the individual components for $N_v = N_c = 2$, $N_k = 300$.

transition rate, the higher is the number of k points required to converge the plot. It can be seen that the rates oscillate as they convergence; if these oscillations are smaller than the final rate, it converges quickly. Otherwise, for smaller rates, these oscillations can be of the same order of magnitude or higher, preventing the rate from converging until they are sufficiently dampened. For $N = 14$, we see that around $N_k \sim 800$ the rates are properly converged, and thus is the baseline we have taken in the calculations shown in the main text. If the width of the ribbon, or the different parameters of the model change, the rates might diminish even further, in which case more k points are added to the calculations.

Finally, we show the plots of the main text but for $N_v = N_c = 2$, for both $Q = 0$ and $Q \neq 0$. While the results are slightly different quantitatively, the same discussion holds. We observe that in presence of the edge offset potential, a charge imbalance can be created at the edges of the material from the dissociation of excitons into edge electron-hole pairs. Analogously, for finite Q a net current can form at the edges of the material as seen from all the transition rates. In the same way, it is possible to engineer the different parameters of the system to tune the rates.

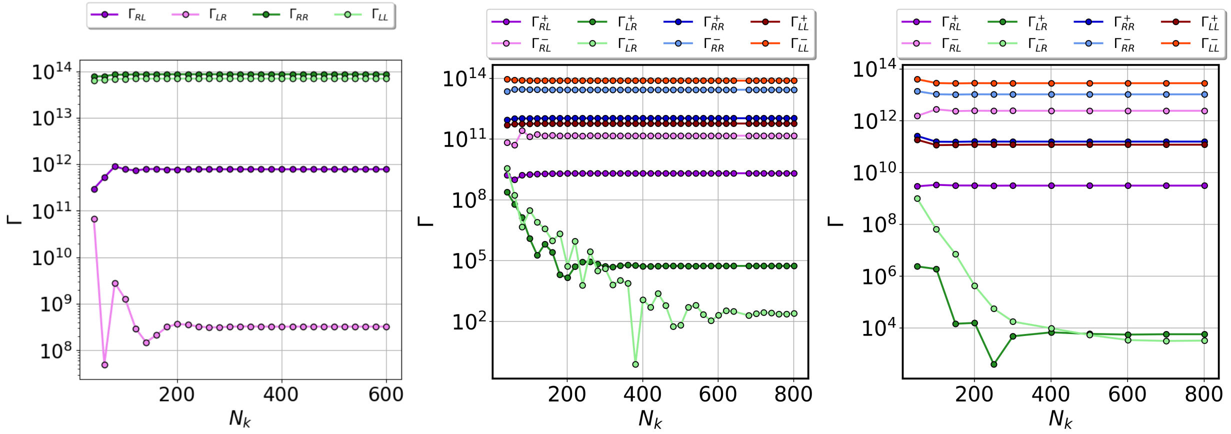

FIG. S8. Convergence of the transition rates $\Gamma^{\pm}_{ss'}$ with N_k. (Left) Rates for a ribbon of width $N = 12$, with $N_v = N_c = 2$ and $Q = 0$. (Middle) Rates for a ribbon of width $N = 14$, for $N_v = N_c = 2$ and $Q = 0.1$. (Right) Transition rates for a ribbon of width $N = 14$, for $N_v = N_c = 4$ and $Q = 0.1$.

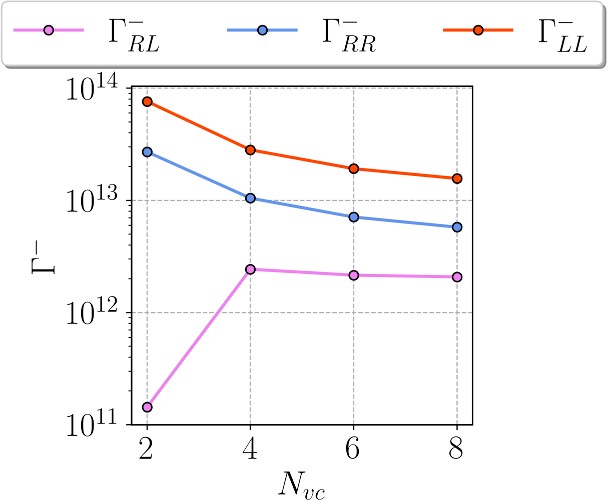

FIG. S9. Convergence of the relevant transition rates with the number of bands N_v, N_c included in the exciton calculation for $N = 14$, $N_k = 200$. We can see that $N_{vc} = 4$ is the minimum number of bands required to ensure that the ratios between the different rates do not change drastically.

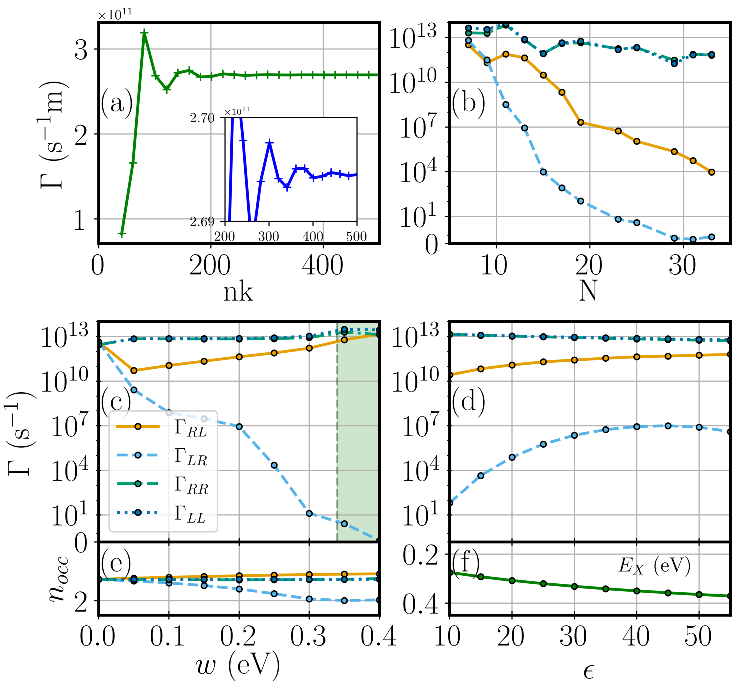

FIG. S10. **Transition rates at $Q = 0$ for $N_v = N_c = 2$.** (a) Convergence of Γ_{LR} for $N = 12$ as a function of the number of points in the BZ. (b) Interedge (Γ_{RL}, Γ_{LR}) and intraedge (Γ_{RR}, Γ_{LL}) transition rates as a function of the number of dimers N of the ribbon. (c) Transition rates with $N = 13$, $\epsilon = 40$ as a function of the edge onsite potential w. The green region indicates that the allowed electron-hole pairs are hosted in different bands. (d) Transition rates with $N = 13$, $w = 0.2$ eV as a function of the dielectric constant ε. (e) Edge occupation n_{occ} of the electron-hole pairs defined as the sum of the electron and hole occupations, as a function of the onsite potential. (f) Exciton energy E_X as a function of the dielectric constant. (b, c, d, e) share the same legend.

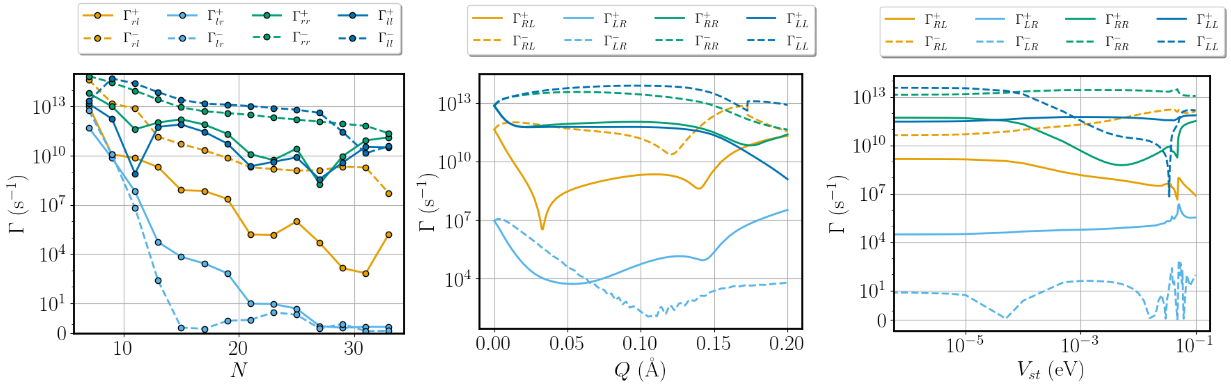

FIG. S11. **Transitions rates for finite Q, for $N_v = N_c = 2$.** (Left) Transition rates as a function of the width of the ribbon, for $Q = 0.1$. All points have been calculated with $N_k = 1200$. (Middle) Transition rates as a function of Q in a ribbon of width $N = 13$. All transition rates are calculated with $N_k = 800$. (Right) Transition rates as a function of the staggered potential V_{st} for $Q = 0.1$ and $N = 13$.

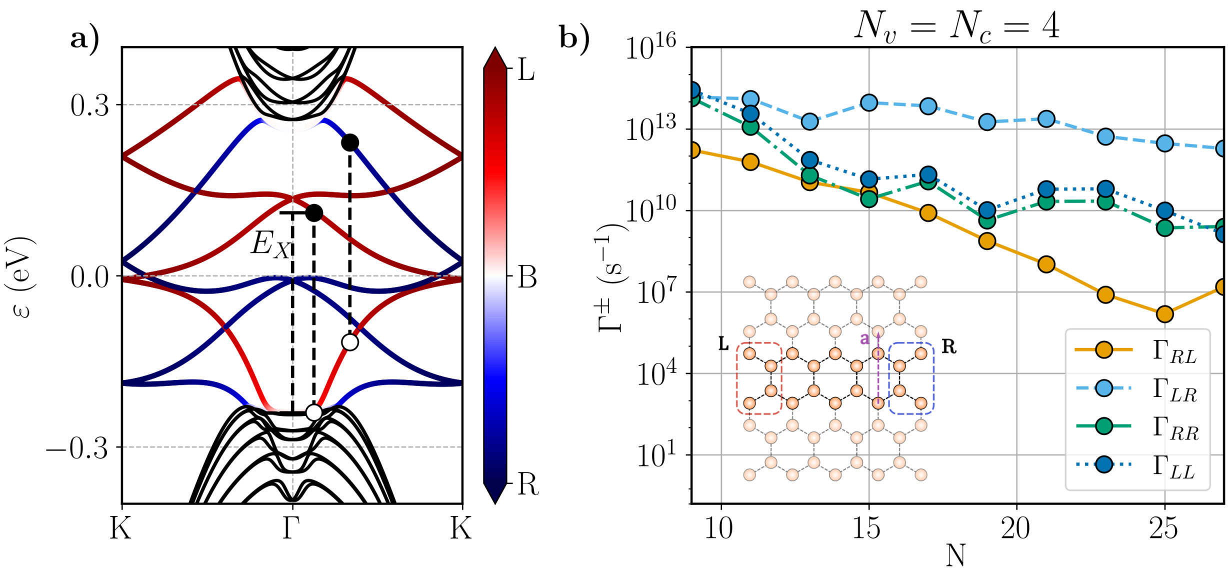

FIG. S12. Rates on an armchair nanoribbon. (a) Band structure of an armchair nanoribbon with an of width $N = 20$ and edge offset of $w = 0.2$ eV. N is an integer such that the unit cell has $4N + 2$ atoms. (b) Transition rates from the bulk excitons to the available edge electron-hole pairs as a function of N, for $N_{vc} = 4$, $Q = 0$ and $N_k = 901$.

For completeness, we also study the effect of the edge termination of the ribbon on the transition rates. The main effect stems from the different band structure, which for the armchair termination results in more complex edge bands. As shown in Fig. S12(a), for the armchair ribbon there are 4 edge bands, which in presence of the edge offset potential results in 8 different bands. Therefore, for one given rate $\Gamma^{\pm}_{ss'}$ with fixed sides s, s' there can be multiple electron-hole pairs available. For simplicity, we only compute the transition rates to the electron-hole pair that gives the dominant contribution to each rates, namely the pairs closer to the Γ point. These rates are shown in Fig. S12(b) as a function of the width of the armchair nanoribbon for $Q = 0$ and $N_{vc} = 4$. We observe the same behaviour as with the zigzag, except for the fact that the domining rate now is Γ_{LR} instead of Γ_{RL}. This is attributed precisely to the more complex band structure, since for the exciton energies present there are two available pairs for the Γ_{LR} rate: one closer to K and another one closer to Γ in the BZ (the latter shown in Fig. S12(a)), while in the zigzag ribbon there was only one close to K. Thus, even though the dissociation direction is reversed, the charge separation mechanism still takes place. Likewise, if we were to consider the rates as a function of Q, we

would expect the same behaviour as in the zigzag, and more importantly, the same direction for the currents given the slope of the bands for the relevant edge electron-hole pairs.

- Proof of $\Gamma^+_{ss'} = \Gamma^-_{ss'}$ for $Q = 0$

We assume that the ground state exciton consists of a degenerate subspace of N states $\{|X_n\rangle\}_n$. Then, since those excitons have $Q = 0$, the time-reversal operator $\mathcal{T}$ will map those excitons onto themselves, i.e.:

$$\mathcal{T}\,|X_n\rangle = U_{nm}\,|X_m\rangle \tag{21}$$

where U is the unitary matrix of the representation. Then, with the transition rate defined as the sum over all degenerate states, we may use that the interaction is time-reveral invariant, $[V, \mathcal{T}] = 0$ to prove the identity:

$$\begin{aligned}
\Gamma^+_{ss'} &= \sum_n |\langle X_n|V|s,s',+k\rangle|^2\,\rho(E_X) = \sum_n \left|\langle X_n|\mathcal{T}^\dagger V\mathcal{T}|s,s',+k\rangle\right|^2 \rho(E_X) \\
&= \sum_n \langle X_n|\mathcal{T}^\dagger V\mathcal{T}|s,s',+k\rangle\,\langle s,s',+k|\mathcal{T}^\dagger V\mathcal{T}|X_n\rangle\,\rho(E_X) \\
&= \sum_{m,p} \underbrace{\sum_n U^*_{nm} U_{np}}_{\delta_{mp}} \langle X_m|V|s,s',-k\rangle\,\langle s,s',-k|V|X_p\rangle\,\rho(E_X) \\
&= \sum_m |\langle X_m|V|s,s',-k\rangle|^2\,\rho(E_X) = \Gamma^-_{ss'}
\end{aligned} \tag{22}$$

- Estimation of the photocurrent produced

We can estimate the current with $j = \eta n(Q)ev$, where $n(Q)$ is the linear density of excitons available for dissociation, η is the efficiency of the effect, e the charge and v the velocity of the final electron once the exciton has dissociated. First, we assume that the linear density of excitons available for dissociation is steady and that the laser is intense enough so that there is always at least one exciton available in the bulk for dissociation. Then, the efficiency η is defined as $\eta = \Gamma^-_{RL}/\sum_{ss'}\Gamma^\pm_{ss'}$, where Γ^-_{RL} is the dominating rate relative to the edge current generation. Thus, $\eta n(Q)$ is the fraction of edge electron-hole pairs relevant to the effect. Since only one exciton can dissociate at a time (since the edge bands are saturated if there is already one electron-hole pair), we set $n(Q) = 1$. Doing the calculation for $N = 14$, $Q = 0.1$ as in Fig. 5, we estimate a current of $j \approx 0.5\ \mu A$. Summing over Q would yield a higher current, but again in the order of μA.

[1] S. Murakami, Quantum spin hall effect and enhanced magnetic response by spin-orbit coupling, Phys. Rev. Lett. **97**, 236805 (2006).

[2] Z. Liu, C.-X. Liu, Y.-S. Wu, W.-H. Duan, F. Liu, and J. Wu, Stable nontrivial Z_2 topology in ultrathin bi (111) films: A first-principles study, Phys. Rev. Lett. **107**, 136805 (2011).

[3] M.-Y. Yao, F. Zhu, C. Q. Han, D. D. Guan, C. Liu, D. Qian, and J.-f. Jia, Topologically nontrivial bismuth(111) thin films, Scientific Reports **6**, 21326 (2016).

[4] C. Sabater, D. Gosálbez-Martínez, J. Fernández-Rossier, J. G. Rodrigo, C. Untiedt, and J. J. Palacios, Topologically protected quantum transport in locally exfoliated bismuth at room temperature, Phys. Rev. Lett. **110**, 176802 (2013).

[5] Y. Liu and R. E. Allen, Electronic structure of the semimetals bi and sb, Phys. Rev. B **52**, 1566 (1995).

[6] R. Gillen, M. Mohr, and J. Maultzsch, Symmetry properties of vibrational modes in graphene nanoribbons, Phys. Rev. B **81**, 205426 (2010).

[7] M. Rohlfing and S. G. Louie, Electron-hole excitations and optical spectra from first principles, Phys. Rev. B **62**, 4927 (2000).

[8] F. Wu, F. Qu, and A. H. MacDonald, Exciton band structure of monolayer mos_2, Phys. Rev. B **91**, 075310 (2015).

[9] M. Bieniek, K. Sadecka, L. Szulakowska, and P. Hawrylak, Theory of excitons in atomically thin semiconductors: Tight-binding approach, Nanomaterials **12**, 10.3390/nano12091582 (2022).

[10] M. L. Trolle, G. Seifert, and T. G. Pedersen, Theory of excitonic second-harmonic generation in monolayer mos_2, Phys. Rev. B **89**, 235410 (2014).

[11] M. F. C. M. Quintela, J. C. G. Henriques, L. G. M. Tenório, and N. M. R. Peres, Theoretical methods for excitonic physics in 2d materials, physica status solidi (b) **259**, 2200097 (2022), (accessed 2024-05-17)., https://onlinelibrary.wiley.com/doi/pdf/10.1002/pssb.202200097.

[12] J. J. Esteve-Paredes and J. J. Palacios, A comprehensive study of the velocity, momentum and position matrix elements for Bloch states: Application to a local orbital basis, SciPost Phys. Core **6**, 002 (2023).

[13] A. J. Uría-Álvarez, J. J. Esteve-Paredes, M. García-Blázquez, and J. J. Palacios, Efficient

computation of optical excitations in two-dimensional materials with the xatu code, Computer Physics Communications **295**, 109001 (2024).

[14] E. Ridolfi, P. E. Trevisanutto, and V. M. Pereira, Expeditious computation of nonlinear optical properties of arbitrary order with native electronic interactions in the time domain, Phys. Rev. B **102**, 245110 (2020).

[15] G. Cistaro, M. Malakhov, J. J. Esteve-Paredes, A. J. Uría-Álvarez, R. E. F. Silva, F. Martín, J. J. Palacios, and A. Picón, Theoretical approach for electron dynamics and ultrafast spectroscopy (edus), Journal of Chemical Theory and Computation **19**, 333 (2023), pMID: 36480770, (accessed 2024-05-17)., https://doi.org/10.1021/acs.jctc.2c00674.

[16] N. S. Rytova, Screened potential of a point charge in a thin film, arXiv e-prints [cond-mat.mes-hall] , arXiv:1806.00976 (2018), (accessed 2024-17-05).

[17] L. V. Keldysh, Coulomb interaction in thin semiconductor and semimetal films, Soviet Journal of Experimental and Theoretical Physics Letters **29**, 658 (1979).

[18] J. Deslippe, G. Samsonidze, D. A. Strubbe, M. Jain, M. L. Cohen, and S. G. Louie, Berkeleygw: A massively parallel computer package for the calculation of the quasiparticle and optical properties of materials and nanostructures, Computer Physics Communications **183**, 1269 (2012).

[19] E. Prada, J. V. Alvarez, K. L. Narasimha-Acharya, F. J. Bailen, and J. J. Palacios, Effective-mass theory for the anisotropic exciton in two-dimensional crystals: Application to phosphorene, Phys. Rev. B **91**, 245421 (2015).

[20] C. Kittel, *Introduction to solid state physics* (John Wiley & sons, inc, 2005).

[21] L. Elcoro, B. Bradlyn, Z. Wang, M. G. Vergniory, J. Cano, C. Felser, B. A. Bernevig, D. Orobengoa, G. de la Flor, and M. I. Aroyo, Double crystallographic groups and their representations on the Bilbao Crystallographic Server, Journal of Applied Crystallography **50**, 1457 (2017).
